# Priorities, barriers, and facilitators for nutrition-related care for autistic children: a qualitative study comparing interdisciplinary health professional and parent perspectives

**DOI:** 10.3389/fped.2023.1198177

**Published:** 2023-08-15

**Authors:** Rachel E. Blaine, Kevin P. Blaine, Katie Cheng, Cynthia Banuelos, Aaron Leal

**Affiliations:** ^1^ Department of Family and Consumer Sciences, California State University, Long Beach, CA, United States; ^2^ Research Institute, Children’s Hospital of Orange County, Orange, CA, United States

**Keywords:** autism spectrum disorder, nutrition, obesity, selective eating, interdisciplinary education, pediatricians, registered dietitian nutritionists

## Abstract

**Introduction:**

Children with autism spectrum disorder often face nutrition-related challenges, such as food selectivity, gastrointestinal issues, overweight and obesity, and inadequate nutrient intake. However, the role of routine nutrition-related screening or care by interdisciplinary health professionals is not well understood. This study aimed to compare the beliefs of health professionals with those of parents of autistic children regarding high-priority nutrition-related challenges, barriers and facilitators to care, and desired education and resources related to nutrition for autistic children.

**Participants:**

Interdisciplinary health professionals (*n* = 25) (i.e., pediatricians, occupational therapists, speech-language pathologists, board certified behavior analysts, registered dietitians) and parents of autistic children (*n* = 22).

**Methods:**

The study used semi-structured phone interviews, which were recorded, transcribed, verified, and double-coded using the Framework Method.

**Results:**

Thematic analysis of transcripts revealed that while health professionals and parents of autistic children shared some perspectives on nutrition-related challenges and care, they also had distinct viewpoints. Parents emphasized the importance of addressing food selectivity, behavioral eating challenges, sensory issues, and sleep disturbances affecting appetite. Both groups acknowledged the need for tailored support, access to an interdisciplinary care team, and reasonable expectations. Some health professionals perceived parents as lacking motivation or the ability to make changes. In contrast, many parents felt that health professionals lacked the knowledge and motivation to take nutrition or growth concerns seriously. Health professionals acknowledged that their lack of knowledge or capacity to provide nutrition education or referrals was a common barrier to care, particularly given limited community resources.

**Discussion:**

Health professionals who serve autistic children are motivated to address nutrition-related challenges but lack resources related to nutrition. To promote better health outcomes for autistic children, professionals should identify and support parent motivations around nutrition-related care. Both groups expressed interest in accessing autism-specific resources for education, referral, and screening guidance. Future research could explore the development of healthcare training models that improve the competency of health professionals in providing nutrition care and referral for autistic children.

## 1. Introduction

Autism spectrum disorder (ASD) is a complex neurodevelopmental disorder characterized by persistent behavioral, communication, and social interaction challenges (1). In the United States, 1 in 36 children is diagnosed with ASD (2). Research shows that 70%–80% of autistic children will experience eating or nutrition-related challenges, including food selectivity, behavioral difficulties with eating, inadequate nutrient intake, and gastrointestinal issues (3–5). The underlying reasons for feeding challenges are multi-faceted and complex, which can make addressing them difficult. For example, many autistic children will experience developmental delays that can impact swallowing, chewing, and the ability to feed themselves independently. Additionally, eating is a full-sensory experience, requiring acceptance of numerous tastes, textures, smells, and colors, all of which can be challenging for children with differences in sensory modulation (6). Finally, autistic children prefer sameness, which makes accepting new and fresh foods problematic as they vary in seasonality, preparation, and brand (5).

Since good nutrition status is essential for growth, learning, attention, and cognition, health professionals must prioritize assessment early and address nutrition-related problems upon identification (7). Research suggests that autistic children who exhibit food selectivity face an increased risk of inadequate nutrient intake for fiber, vitamins A, C, D, and E, calcium, zinc, and folic acid (8–10). Despite experiencing food selectivity, autistic children are significantly more likely to be overweight or obese than neurotypical peers. They are subsequently at higher risk for experiencing health disparities related to chronic diseases like diabetes and heart disease as they enter young adulthood (11, 12).

Providing comprehensive care for children with Autism Spectrum Disorder (ASD) involves the collaboration of various interdisciplinary medical professionals, including developmental-behavioral pediatricians, board-certified behavior analysts, occupational therapists, speech-language pathologists, and registered dietitian nutritionists. However, there is a limited understanding of how these medical professionals prioritize nutrition-related issues in their practice. Competence in addressing nutrition challenges in autism likely varies across disciplines.

For example, one study found that a majority of graduating medical students rated their nutrition knowledge as inadequate (13). Since nutrition education is often insufficiently incorporated into medical school curricula, a lack of familiarity with nutrition-related topics may impact pediatricians’ confidence and effectiveness in providing evidence-based nutrition care to parents and patients (14). Similarly, other allied health professionals involved heavily in feeding therapies (i.e., occupational therapists, and behavioral analysts) also often lack specific nutrition-focused training. One study indicated gaps in nutrition training for speech-language pathologists and occupational therapists primarily due to a lack of implementation in educational settings. Consequently, there are limitations in their collaborative roles within nutrition competencies (15).

These findings highlight the need for improved nutrition education and training across medical and allied health disciplines involved in caring for children with ASD. Addressing these gaps can enhance the overall competence and effectiveness of medical professionals, ensuring the provision of holistic and evidence-based nutrition care to improve the health outcomes of children with ASD (12, 16). Examples of nutrition-related care could include any intervention (e.g., screening, education, referrals) for factors directly or distally related to dietary intake, growth, or nutrient absorption, including impacts on appetite (e.g., sleep, physical activity), gastrointestinal issues, medication, and anxiety around eating.

To address the existing gaps in the current body of literature, we conducted an exploratory qualitative study to better understand the views of interdisciplinary health professionals and parents of autistic children. Our three primary objectives were to: (1) describe perceived nutrition-related challenges that autistic children face, (2) identify barriers and facilitators to nutrition-related care, and (3) identify desired educational topics and resources for nutrition-related care for autistic children. Across all three objectives, we explored shared and differing perspectives between medical professionals and parent participants.

To ensure a systematic approach to our methods and analysis, we utilized the Theoretical Domains Framework. This evidence-based and theory-based framework enabled us to evaluate the practices of health professionals and their experiences with the healthcare system, identify potential areas for implementation, and enhance the quality of care. By considering cognitive, affective, social, and environmental influences on behavior, the Theoretical Domains Framework provided valuable guidance for our methods and analysis. Our research findings hold significant implications for developing future interventions aimed at improving nutrition and growth outcomes for children with ASD.

## 2. Methods

### 2.1. Participants

Our study aimed to recruit a minimum of 20 pediatric-focused health professionals who also treated patients with autism and 20 parents of autistic children. These sample sizes were deemed appropriate for a study using semi-structured interviews based on existing qualitative research best practices to achieve thematic saturation (17). By employing a combination of convenience purposive criterion sampling and nominated expert sampling, we selected a sample of interdisciplinary professionals involved in autism care across various professions and practice areas, focused on serving a pediatric population. We recruited no more than five health professional participants per profession across six distinct professions, which included general pediatricians, developmental-behavioral pediatricians, occupational therapists, speech-language pathologists, board-certified behavior analysts, and registered dietitian nutritionists.

Health professionals were recruited using internet searches, infographic flyers, and referrals from other interviewees to identify potential participants. We opted to use nominated expert sampling for pediatricians who were especially difficult to recruit during the COVID-19 pandemic (18). After voluntary acceptance, each health professional completed an interest screener survey to determine eligibility. Health professionals were eligible to participate if they had active status in their profession, had at least a year of experience working with children on the autism spectrum, and currently practiced in California.

Parents were recruited via flyers at local non-profit Regional Centers and on two Southern California Facebook autism parenting support groups. To address the lack of perspectives from people of color and fathers in the literature, we intentionally sought to achieve a racially and ethnically diverse group of parents, including both mothers and fathers (19, 20). Eligible parents were English-speaking, the primary caregiver of an autistic child between the ages of 3–18 years, and primarily responsible for feeding their child. If a parent had more than one autistic child, they were instructed to focus on their youngest child for this study.

After a brief screening process, eligible participants were contacted via phone or email to schedule an interview. They were then emailed confirming the interview appointment, an electronic consent form, and a brief pre-interview demographics questionnaire administered online via Qualtrics. All enrolled participants provided written informed consent and received a $25 Amazon gift card as compensation for participating in the study. The Institutional Review Board at California State University, Long Beach, reviewed and approved all aspects of this study.

### 2.2. Interview guides

Two separate semi-structured interview guides for health professionals and parents were developed as part of a broader study seeking to develop a nutrition-related assessment screening tool for children on the autism spectrum. Both guides consisted of open-ended questions and were developed based on previous research and informed by the Theoretical Domains Framework of behavior change (21). The guides can be found in Supplementary Data Sheet S1.

The Theoretical Domains Framework is a widely used behavior change framework that considers various factors influencing healthcare professionals’ clinical behavior change (22). These factors include motivation, capability, and opportunity. We used this framework to guide the development of interview questions and to assess participants’ views on nutrition-related risk factors for children with autism. To guide the development of our interview guide, we identified three content matter experts who were all experienced researchers and registered dietitians with extensive clinical practice experience with children with ASD. The experts reviewed multiple iterations of the two interview guides and provided feedback to promote face validity.

Before the interview, both groups of participants were given a list of nutrition-related challenges for children with autism and asked to review it. Nutrition-related challenges were defined as any health indicators or lifestyle factors directly or distally related to dietary intake, growth, or absorption, including impacts on appetite (e.g., sleep, physical activity), gastrointestinal issues, medication, and anxiety around eating. During the interview, they were asked to identify which items they felt were most important on that list (motivation), about their experiences with nutrition-related care/conversations (opportunity), and about other resources or support they felt would help address these challenges (capability). This list of challenges was also informed by our content matter experts.

### 2.3. Procedures

Interviews were conducted over the phone between October 2019 and June 2020, with each interview ranging from 15 to 30 min. Two graduate students (C.B., K.C.) and one undergraduate research assistant (A.L.) led the interview using the interview guides; each was trained in qualitative methods by R.B. before commencing the first interview. Afterward, interviewers completed field notes using a specific template to document environmental, methodological, and analytic observations and the overall quality grade of the interview data collected. All interviews were audio-recorded with two devices and stored securely.

### 2.4. Analytic approach

To analyze our qualitative data, we used the Framework Method, which is a combined inductive and deductive approach to thematic qualitative analysis (23). The deductive component uses an evidence-based theoretical framework to explore specific issues and thus begins with a coding scheme. However, the Framework Method also allows for inductive analysis through open, unrestricted coding and refinement of emergent themes. For Objective 1, describing perceived nutrition-related challenges that autistic children face, and Objective 3, identifying desired educational topics and resources, we used an inductive approach through open, unrestricted coding and refinement of emergent themes identified by both groups of participants.

For Objective 2, identifying barriers and facilitators to nutrition-related care, we used the Theoretical Domains Framework to organize the main themes of barriers and facilitators across pre-defined domains of motivation, capability, and opportunity that influence healthcare professionals’ nutrition-related care and conversations with parents of children on the autism spectrum (22). We selected this deductive approach to allow for the organization of findings using a theory-based lens for understanding clinical behavior and behavior change with the hope that this organization would lead to clearer formative data to inform future interventions. Finally, we planned to explore shared and divergent perspectives between medical professionals and parents using coding matrices.

### 2.5. Data analysis

Following the completion of the interview, each recording was transcribed verbatim, de-identified, and verified for accuracy by the original interviewer and another researcher. An initial codebook was tested by three reviewers who coded ten randomly selected transcripts (CB, KC, RB), ensuring intercoder agreement through weekly meetings, discussion, revision, and consensus on newly identified emergent subthemes using constant comparative methods. Emergent themes were discussed, discrepancies reviewed, and finalized codes were synthesized into the next iteration of the codebook. When new emergent codes were added, the researchers re-coded the prior transcripts using the latest coding schema. Twelve additional transcripts were double coded with the revised codebook and with ongoing review and iterative revision. Finally, two researchers (CB, KC) reviewed all former and remaining transcripts using the finalized codebook and demonstrated good inter-rater reliability (_K_ coefficient between 0.75 and 0.8). The final codebook can be found in Supplementary Data Sheet S2. The team determined that data saturation had been reached when no new emergent codes were identified in the last five coded transcripts of both health professional and parent groups.

Finally, we ran two types of matrix queries based on participant characteristics to identify whether or not there were differences in the frequency of subthemes between groups. The first matrices compared subthemes between health professionals and parents. The second matrices examined subthemes between health professionals based on their self-reported frequency of exposure to clients with ASD. We identified the cutoff using a median split of high exposure (≥45% of patients with ASD) and low exposure (<45% of patients with ASD). All thematic analyses were performed using NVivo version 12. Demographic data were analyzed using Microsoft Excel to obtain frequencies, means, and standard deviations to describe the participant populations.

## 3. Results

Forty-seven participants completed in-depth interviews for this study. Health professional participants (*n* = 25) were predominantly female (76%) and identified as White (48%), Asian (28%), or Multiracial (16%). Their estimated years of experience working with children with ASD ranged from 3 to 34, with a mean of 10.7 years (SD = 8.7). Participants included pediatricians, developmental-behavioral pediatricians, occupational therapists, speech-language pathologists, board-certified behavior analysts, and registered dietitian nutritionists. They worked primarily in outpatient medical facilities (44%), private practice (24%), and school districts (20%). Only a minority (24%) were part of an interdisciplinary team that included a registered dietitian nutritionist. Additional health professional demographic details are presented in Table 1.

**Table 1:** Demographic characteristics of *n* = 25 health professionals with experience serving children diagnosed with autism spectrum disorder (ASD).

Demographic characteristics	*n*/mean	%/SD^a^
Total participants	25	
Gender identity		
Female	19	76
Male	5	19
Race and ethnicity^b^		
White	12	48
Multiracial	4	16
Asian	7	28
Black/African American	0	0
Other	2	8
Latino/Latinx/Hispanic	3	11
Profession		
Pediatrician (general)	4	16
Pediatrician (developmental-behavioral)	3	12
Occupational therapist	5	20
Speech-language pathologist	4	16
Board certified behavior analyst	5	20
Registered dietitian nutritionist	4	16
Work setting		
Outpatient medical facility	11	44
Private practice	6	24
School district	5	20
In-home	2	8
Other	1	4
Part of an interdisciplinary team with a registered dietitian	6	24
Years of experience as health professional (mean, SD)	11.4	10.6
Years of experience working with children with ASD (mean, SD)	10.7	8.7

^a^ SD, standard deviation.

^b^ Participants chose all that applied.

Parent participants (*n* = 22) were also primarily female (82%) and identified as Latino/Latinx/Hispanic (50%) or White (33%). Fewer than half were employed, and 41% were out of the workforce to care for a child or parent. The mean age of the children described was 6.8 years (range: 3–15 years), and the majority were male (82%). Additional parent demographic details are presented in Table 2.

**Table 2:** Demographic characteristics *n* = 22 parents and their child with a diagnosis of autism spectrum disorder (ASD).

Demographic characteristics	*n*/mean	%/SD^a^
Total participants	22	
Parent characteristics		
Gender identity		
Female	18	82
Male	4	18
Relationship to child		
Mother	18	82
Father	4	18
Race and ethnicity^b^		
White	8	33
Multiracial	1	4
Asian	1	4
Black/African American	1	4
Other	1	4
Latino/Latinx/Hispanic	11	50
Highest level of education		
High school graduate or GED^c^	1	5
Technical school	3	14
Some college	6	27
College graduate	6	27
Graduate or professional degree	6	27
Employment status		
Employed for wages	10	45
Out workforce to care for a child/parent	9	41
Full-time student	2	9
Self-employed	1	5
Age in years (mean, SD)	37	6.4
Number of children in household (mean, SD)	1.7	0.5
Reference child characteristics		
Gender		
Female	4	18
Male	18	82
Age in years (mean, SD)	6.8	3.4

^a^ SD, standard deviation.

^b^ Participants chose all that applied.

^c^ GED, General education development.

### 3.1. Group-level differences

We did not observe differences in subtheme frequency between health professionals who worked more frequently with patients with ASD versus those less frequently across any of the coding domains. Therefore, our group-level findings will be based on the differences and similarities between groups of health professionals and parents of autistic children which were observed across all main themes and subthemes.

### 3.2. Nutrition-related challenges for autistic children

All health professionals interviewed reported encountering nutrition-related challenges while working with patients on the autism spectrum. Similarly, most parents (90%) voiced ongoing nutrition-related challenges for their children, with all having experienced challenges in the past. When asked to identify which nutrition-related challenges should be prioritized and addressed through referral or direct education, subthemes were organized by emergent among both groups, only health professionals, and only parents. Table 3 summarizes key themes, subthemes, and illustrative quotes.

**Table 3:** Themes with illustrative quotes regarding nutrition-related challenges to address for autistic children.

Theme: nutrition-related challenges to address for autistic children	
Shared subthemes: health professionals and parents	Illustrative quotes
Obesity	Well, he went up a size every 2 months for a year. So financially that was intense, and it was also really scary for me just when is he going to stop growing or slow down. (Mother to 6-year-old boy, ID 110)
	With obesity and prediabetes and diagnosis of diabetes, because that’s huge in terms of impacting their mortality. (Board Certified Behavior Analyst, ID 219)
Underweight	If their growth is really significantly delayed or a big change from what they've been doing normally, that would be a big red flag for me to refer somebody to a dietitian. (Pediatrician, ID 227)
	If they're diagnosed with failure to thrive, I think that’s high risk, just because failure to thrive is inability to put on weight and shows appetite issues. (Registered Dietitian Nutritionist, ID 213)
Gastrointestinal concerns	So, if a child is having water reflux issues, then I refer them back to the pediatrician to see if they should be medicated for that. (Occupational Therapist, ID 224)
	Every single family that I know with high-functioning kids on the spectrum have the gastrointestinal issues. (Mother to 9-year-old boy, ID 107)
	He has so much gas and his bowel movements were out of control. Mother to 3-year-old boy, ID 136)
Medication weight-related side effects	I know it's the meds that make him not hungry. I hate that side effect. (Mother to 13-year-old boy, ID 135)
	But I've definitely noticed with some clients, when parents do change their medication, it does affect the appetite. So, a lot of times, when they do put on more medication, they do tend to increase in weight. (Board Certified Behavior Analyst, ID 228)
	Well, typically patients who are on atypical antipsychotics are gonna gain weight (Developmental Pediatrician, ID 233)
Subthemes among health professionals	Illustrative quotes
Focus on specific high-risk diagnoses	I think if, say, this child has more complex issues or they have severe allergies to certain types of foods, that would need more attention. (Occupational Therapist, ID 223)
Dental hygiene	A lot of our kiddos don't like to brush their teeth. I think it’s a huge concern. (Board Certified Behavior Analyst, ID 221)
Parent lack of knowledge	If I feel like the parent has a lack of knowledge around food and food groups and portions, then I’ll refer. (Developmental Pediatrician, ID 233)
Oral motor challenges	If they're actually having ongoing problems with feeding, so like choking or gagging while feeding. (Developmental Pediatrician, ID 234)
	Children that have neuromotor issues—so, child with cerebral palsy that may have swallowing dysfunction as a result of that or, or issues with fine motor skills with feeding and chewing—they should get referral to a dietitian as well to address those concerns and screen for any particular problems. (Pediatrician, ID 235)
Subthemes among parents	Illustrative quotes
Poor diet quality	I'm definitely concerned. There's always a fine line between, is his behavior a result of his atypical brain, or is it because he's lacking nutrition because of his picky eating? (Mother to 11-year-old boy, ID 100)
	He’s healthy for his height and weight and his age. But that’s why they don’t really see a concern. But, as a parent, I’m like, wait, he might not be getting in all the food groups in. (Mother to 3-year-old boy, ID 104)
	Look growing, he's fine. That's why I'm not worried about him getting fat right now or anything like that. All he eats is, like I said, pancakes and waffles, chicken strips, won't eat anything else. I'd been worried about that with him not getting enough nutrition (Father to 15-year-old boy, ID 143)
Behavioral feeding challenges	Meltdowns aren't mentioned [around eating] and can be severe. He hurts himself. (Mother to 13-year-old boy, ID 135)
	It’s hard to get him to sit down and eat a meal. (Mother to 3-year-old boy, ID 105)
	It's a constant struggle to make sure he eats, and if he doesn't eat and gets in a bad mood, and then he gets in trouble at school. (Mother to 7-year-old boy, ID 118)
Sensory challenges	I would say the inability to sense fullness, that's kind of like the biggest issue here. (Mother to 8-year-old girl, ID 115)
	Gagging while eating is actually a very big one that I've spoken to a lot of parents lately, because the sensory input of when another child is seeing somebody else eat. Like my son, if he sees somebody chewing with his mouth open, he's done, has to get away, cannot be anywhere near those people. (Father to 9-year-old boy, ID 144)
	He will react very strongly to smells. For example, like the smell of fresh baked bread like if we’re walking by a bakery, he will start kinda like gagging. So, it's hard, you know, in the school setting, he also has to go to the cafeteria or there’s like a party. *(Mother to 6-year-old boy, ID 109)*
	I think a lot of her eating aversions have to do with sensory things. (Mother to 8-year-old girl, ID 116)
Sleep	On the days with difficult sleep there's more of a craving for carbs, you know, and he always wants carbs, but it's definitely more, "Can I have cake, can I have cookies?" (Mother to 6-year-old son, ID 110)
	Ask nine out of 10 parents, yes, our kids don't sleep. (Father to 9-year-old boy, ID 144)

Both professionals and parents identified nutrition-related challenges for autistic children related to obesity, underweight, and gastrointestinal concerns. Additionally, medications were identified as a concern for contributing to both undernutrition and overnutrition.

*I know it’s the meds that make him not hungry. I hate that side effect. (Mother to 13-year-old boy, ID 135).*

*Well, typically patients who are on atypical antipsychotics are gonna gain weight (Developmental Pediatrician, ID 233).*

Health professionals specifically identified four distinct nutrition-related challenges, including high-risk diagnoses (e.g., food allergies, epilepsy), poor dental hygiene, oral motor challenges, and parental lack of nutrition knowledge. Lack of knowledge was cited as contributing to poor diet quality and parenting strategies. There were no differences in subthemes between professionals who worked more versus less frequently with autistic children.

One distinct subtheme that emerged from the parent group was the importance of addressing poor diet quality, even among children with average growth. Many expressed anxiety and concern due to extreme food selectivity.

*Look growing, he’s fine. That’s why I'm not worried about him getting fat right now or anything like that. But all he eats is, like I said, pancakes and waffles, chicken strips, won't eat anything else. I'd been worried about that with him not getting enough nutrition. (Father to 15-year-old boy, ID 143).*

*I'm definitely concerned. There’s always a fine line between, is his behavior a result of his atypical brain, or is it because he’s lacking nutrition because of his picky eating? (Mother to 11-year-old boy, ID 100).*

Additionally, parents described the need to address behavioral feeding challenges that affect quality of life in addition to dietary intake, as well as sensory challenges impacting mealtime enjoyment. They expressed the need for behavioral feeding strategies to improve their children’s overall quality of life, including sleep.

*Meltdowns aren't mentioned [around eating] and can be severe. He hurts himself. (Mother to 13-year-old boy, ID 135).*

*He will react very strongly to smells. For example, like the smell of fresh baked bread, like if we’re walking by a bakery, he will start kinda like gagging. So, it’s hard, you know, in the school setting, he also has to go to the cafeteria, or there’s like a party. (Mother to 6-year-old boy, ID 109).*

*On the days with difficult sleep there’s more of a craving for carbs, you know, and he always wants carbs, but it’s definitely more, “Can I have cake, can I have cookies?” (Mother to 6-year-old son, ID 110).*

*Ask nine out of 10 parents, yes, our kids don't sleep. (Father to 9-year-old boy, ID 144).*

It is worth noting that physical activity was rarely identified as a nutrition-related priority by either group, despite its well-established role in improving quality of life, mental health outcomes, and weight balance (24, 25).

### 3.3. Barriers and facilitators to nutrition-related care

Barriers and facilitators to care for nutrition-related challenges for autistic children were categorized into three domains: motivation, capability, and opportunity, based on the Theoretical Domains Framework (22). A summary of key themes and subthemes, organized by domain, can be found in Table 4, with a complete table of subthemes and illustrative quotes in Supplementary Table S1.

**Table 4:** Summary of themes regarding barriers and facilitators to care for nutrition-related challenges for autistic children.

Domain^a^	Shared subthemes: health professionals and parents	Subthemes among health professionals	Subthemes among parents
Theme: barriers to care for nutrition-related challenges			
**Motivation** *Beliefs, optimism, goals*	• Competing priorities for families	• Lack of parent compliance • Change may not be possible • Concerns that interventions worsen eating challenges	• Concerns not taken seriously • Judgment from providers
**Capability** *Knowledge, behavioral skills*	• Lack of autism-relevant information • Providers unsure how to help	–	• Child’s growth in normal range
**Opportunity** *Environmental context and resources*	• Lack of access to nutrition services • Limited time during visits • Difficult doing nutrition education with child present • Family financial limitations	• Limited referral options	• Unaware of how to navigate services
Theme: facilitators to care for nutrition-related challenges			
**Motivation** *Beliefs, optimism, goals*	–	• Realistic expectations for setting goals	• Other parents of autistic children offer support
**Capability** *Knowledge, behavioral skills*	• Individualized support	• Embracing slow progress	• Mental health professionals offer support • ABA therapy helpful for feeding
**Opportunity** *Environmental context and resources*	• Access to an interdisciplinary team	• Nutrition-related screening part of practice	–

^a^ Based on the theoretical domains framework of behavior change.

#### 3.3.1. Motivation-related barriers to care

In the Theoretical Domains Framework, motivation encompasses internal factors that impact behavior (e.g., beliefs, optimism, goals). A total of six subthemes emerged as motivation-related barriers to care for nutrition-related challenges for autistic children. Both healthcare professionals and parents shared one subtheme: *competing priorities for families,* identified by most participants in both groups. Examples of competing priorities included parents managing many appointments and medical therapies for their children and overall burnout when facing a child’s more severe emotional or behavioral problems.

*So what happens is a lot of the things can be lost in translation because the parents are tired. They have a lot going on. They work, they have the kids. (Registered dietitian nutritionist, ID 217).*

*Honestly, we had other more behavioral concerns to address. It [his growth] just wasn't a priority to me. (Mother to 10-year-old boy, ID 119).*

Among health professionals, three subthemes emerged, centered on either parent or child motivation: (1) *lack of parent compliance*, (2) *change may not be possible*, and (3) *concern that intervention worsens eating challenges*. Many professionals expressed concerns that some children were simply too resistant to change and, thus might not successful.

*If we were trying to increase activity or we were trying to change intake to foods that were lower in calories or healthier choices, it was one thing to recommend it, and it was another thing to actually have it take place. Mainly because the child would be unwilling to do it. (Pediatrician, ID 225).*

*I also see kids that will very repetitively eat the same thing all the time, and I know that it’s unhealthy for them instinctually, but it’s hard to do anything about it because they're so entrenched in their schedule and their diet. (Occupational Therapist, ID 223).*

Some health professionals thought parents avoided addressing nutrition or diet issues because they were concerned that intervention would cause more harm than good.

*I think the concern for a lot of parents, too, is like they're wary of working on it because they don't want their kiddo to like stop eating everything altogether. And they're eating really unhealthy foods, but at least they're eating you know. (Board Certified Behavior Analyst, ID 221).*

*Parents are afraid that if they don't give the kids what they know that they'll eat, that the kids will not be gaining weight. That further restricts their diets or further narrows their diets. (Pediatrician, ID 222).*

The two main barrier subthemes among the parent group focused on medical professionals’ motivation. Parents identified (1) *concerns not taken seriously* and (2) *provider judgment* as barriers to their child’s care. Many parents reported that professionals were unmotivated to address nutrition-related concerns, despite parents expressing a desire to.

*Because one, most doctors, and I hate to say it like this, but can be very condescending to a patient’s requests or concerns. Yeah, they see the child for five minutes in the appointment, but we, as the parents, live with the child 24/7. The parent input is very important, and the concerns need to be addressed. (Father to 9-year-old boy, ID 144).*

Some discussed conversations with professionals that made them feel judged, leading parents to feel unsupported.

*The general pediatrician has not been very receptive. He tends to brush these things off. Sometimes I feel that in discussions on picky eating, they tend to be kind of like judgmental, and it falls hard on the parent. (Mother to 6-year-old boy, ID 109).*

#### 3.3.2. Motivation-related facilitators to care

While no shared subthemes for motivation-related facilitators to care emerged across both groups, two main subthemes emerged. The first, *having realistic expectations*, emerged from the health professional group, and centered around reasonable goal-setting with parents. Professionals described how too many or unrealistic goals could inhibit motivation and progress for their patients.

*I think that trying to break it down for parents usually makes it less daunting and makes them more able to grasp and practice what it is we talk about in the office at home. (Pediatrician, ID 222).*

Among the parent group, a critical motivation-related facilitator was *support from other parents of autistic children* to offer motivation for nutrition care. When parents would get discouraged, they often cited other parents as being a source of inspiration and encouragement to continue addressing their children’s challenges.

*Nothing helps outside other than the mommy group talks that we ask each other like, “What works for your kid?” And that’s been helpful. But it’s super common among all our moms that our kids don’t want to eat and have a hard time. (Mother to 3-year-old boy, ID 104).*

#### 3.3.3. Capability-related barriers to care

Capability is defined as having the necessary knowledge and skills required to engage in a behavior. In this case, the behavior could be education or referral for nutrition-related challenges for autistic children. A total of three subthemes emerged as capability-related barriers to care.

The first subtheme centered around *a lack of available autism-relevant information*. Both groups described that many online resources, books, and handouts were geared toward neurotypical children and didn’t always apply to the needs of autistic children.

*I think it’s difficult because even if we have nutrition classes or supplemental information for the parents to look into, very rarely are they specifically for autistic kids. (Pediatrician, ID 227.*

Additionally, both groups suggested that *providers are unsure how to help,* either because providers lack nutrition knowledge or awareness of referral options. Professionals acknowledged limitations in their knowledge and training about nutrition and autism, which was echoed by parents feeling that they, “*haven’t gotten much help”* from their child’s healthcare team (ID 118). One pediatrician explained (ID 225),

*“Most of us didn't get much nutrition education in medical school or residency.”*

A consistent theme from several parent interviews was that *their child’s normal growth* was a barrier to care. Parents indicated that they felt providers lacked the knowledge about the impact and importance of a balanced diet despite a typical growth pattern. This theme repeatedly emerged among parents who expressed significant concerns about selective eating patterns.

*His doctors, they don't seem too concerned because he’s not losing weight. (Mother to 13-year-old boy, ID 135).*

*But I haven't felt a lot of support from the nutrition part because they think he is good cause he’s at a good weight. (Mother to 3-year-old boy, ID 104).*

#### 3.3.4. Capability-related facilitators to care

Four subthemes emerged as capability-related facilitators to care. The first, *individualized support*, was identified across both groups as critical to promoting nutrition care for autistic children. Parents explained the importance of “customized” strategies to address challenges, and providers described the need to consider culture, income status, and the needs of each child/family.

*I'm taking into account the family’s culture, so what their daily routine is like, what their menu, what type of food that they're usually eating and then making sure that the interventions and strategies that I teach and educate the parents with can easily fit it to their everyday routine. (Occupational Therapist, ID 224).*

Health professionals specifically identified the need for *embracing slow progress* when making nutrition-related changes. This requires educating parents about progress expectations and avoiding overwhelming them with too much information.

*Definitely flexibility, patience, the ability to go in at a slow pace and take baby steps and not have expectations that are out of line with what the parent feels they can do. (Speech-Language Pathologist, ID 214).*

Two subthemes that emerged only in the parent group were the specific capabilities of *mental health professionals* and *Applied Behavioral Analysts* in facilitating nutrition-related care. Parents explained that these providers spent more time monitoring lifestyle habits (e.g., eating, sleep, and activity) than others. Specifically, psychiatrists were identified as key facilitators of nutrition-related care among multiple parents, as they were more attentive to weight shifts due to medication management.

*I've gotten the most feedback and understanding from a neuropsychologist. Somebody that has a little bit more understanding of the nervous system itself and sensory relation. And not just, “Oh, it’s anxiety,” or, “He'll outgrow it.” or, “Keep trying.” (Mother to 11-year-old boy, ID 100).*

*One of the psychiatrists he was seeing actually recommended that because I was giving him like a Pedisure, and he’s like, he’s getting bigger. She says give him this because it has more carbs and more protein, and that’s what he needs. (Mother to 13-year-old boy, ID 135).*

#### 3.3.5. Opportunity-related barriers to care

Opportunity is defined as the presence of external factors that make a behavior possible, including environmental context and resources. The greatest number of barrier themes emerged within the Opportunity domain, with six emergent subthemes.

Both groups identified *a lack of access to nutrition services*, primarily related to feeding therapy or registered dietitian nutritionists understanding autism.

*Even if parents are willing and wanting to put in the work, a lot of times, there’s not the service available to guide them. (Pediatrician, ID 222).*

*I can't actually have a dietitian on our team unless our doctor refers us because of our medical insurance. (Mother to 11-year-old boy, ID 100).*

Both groups also described two limitations during visits, which included *limited visit time* and *difficulty doing nutrition education with child present* at a visit.

*A lot of us already have large scopes of practice, and it’s like [nutrition care] that’s such an important aspect, and yet we just don't have time. (Speech-Language Pathologist, ID 218).*

*Having an autistic child with you at the pediatrician’s office, I don’t feel like you have the time to really focus on your answers. I’m not going to retain much because my brain has just been in fight or flight at that point!” (Mother to 4-year-old boy, ID 128).*

Finally, both groups discussed how *financial limitations* impacted access to healthcare services and healthy foods. A few providers explained that feeding therapy requires buying and trying multiple types of foods that can be wasted, which is not always feasible for their patients.

*So, some of it is the resources of just having the money to buy the food or to maybe screen different foods, right? You know, we say, “Hey, why don’t you go buy this, this, this, and this?” (Board Certified Behavior Analyst, ID 220).*

Parents echoed health professionals’ concerns about being able to purchase affordable foods that their provider recommended and that their child would eat, and providers explained that food insecurity also could impact therapeutic sessions.

*And there’s just no good options. A working mom, working single mom to [be able to] give him unprocessed foods that have plenty of nutrition and protein. (Mother to 7-year-old boy, ID 118).*

*A lot of my families too, who are very low income… in not having control or influence on what a kid eats and whether or not they're hungry. That can change the way an entire session with the child will go or an entire day a child will have. (Board Certified Behavior Analyst, ID 219).*

#### 3.3.6. Opportunity-related facilitators to care

Two subthemes emerged as opportunity-related facilitators to care. The first appeared across both groups was *access to an interdisciplinary care team that included a nutrition professional* or therapist with expertise in nutrition. Parents who had access to these types of teams explained how their “team of therapists” gave them access to professionals who could answer their questions. Health professionals also felt access to others across disciplines, especially a registered dietitian, helped promote nutrition care.

*Oh, the registered dietitians on our team, for sure. Yeah, I'm really lucky that we have RDs in-house cause I definitely feel very lost without them. (Occupational Therapist, ID 211).*

*I think it’s really a team approach. I think you have to have a nutritionist who understands what autism is, but you also need to have physicians or developmental-behavioral pediatricians who understand how to understand the child’s unique characteristics. (Developmental Pediatrician, ID 233).*

Finally, a subtheme that emerged only among health professionals was how *nutrition-related screening* could facilitate care by stimulating discussions with families. Typically, this was described in the context of intake among only a few participants, namely pediatricians/developmental pediatricians, registered dietitians, and occupational therapists. Some examples of screening included the regular use of growth charts (i.e., height, weight, body mass index), adaptive skills history, intake forms related to diet or sensory challenges, and conversations with parents about eating issues.

### 3.4. Education opportunities and resource availability

When asked to identify potential educational topics and resources needed to address nutrition concerns for autistic children, participants discussed various topics of interest and delivery platforms. A summary of themes and subthemes can be found in Table 5. A complete table of themes and illustrative quotes can be found in Supplementary Table S2.

**Table 5:** Summary of themes regarding desired educational topics and resources related to nutrition-related challenges for autistic children.

Shared subthemes: health professionals and parents	Subthemes among health professionals	Subthemes among parents
Desired topics for education regarding nutrition-related challenges for autistic children		
• Dietary guidelines specific to autism • Behavioral strategies to address food selectivity • Dietary supplements • Efficacy of specialized diets for autism	• General autism education • Basic nutrition and feeding • Steps to take for providing support	• Meeting dietary needs within a restricted diet • Gut health • Pica
Desired resources to address nutrition-related challenges		
• List of local specialists or providers • Parent-friendly website	• Access to a registered dietitian nutritionist for professional advice • Evidence-based paper materials to supplement conversations	• Community groups

Overall, both groups were interested in learning more about dietary guidelines specific to autism, behavioral strategies to address food selectivity, dietary supplements (e.g., probiotics, fiber), and the efficacy of specialized diets such as gluten-free and casein-free diets, both of which are commonly sought alternative treatments parents turn to for children with ASD with limited evidence (26).

Health professionals—mainly pediatricians—identified interest in learning more about autism generally, while others emphasized learning basic nutrition and feeding recommendations. Professionals were also eager to understand the necessary steps for providing evidence-based nutrition-related support, particularly regarding referrals to other interdisciplinary team members.

*I feel like oftentimes we can address it [nutrition concerns], but we would need access to a dietitian or knowing when we should go to them. (Occupational Therapist, ID 231).*

*Because eating is a behavior, but it’s also internal, and it’s also psychological, and it’s also oral motor. So, there needs to be like a really good cohesive, comprehensive team that’s there to support the child in feeding. (Board Certified Behavior Analyst, ID 219)*

When asked about potential platforms for delivering nutrition education or resources, both groups identified the need for a clear list of relevant resources, including specialty providers with nutrition and feeding expertise.

*A list of local professionals that we can refer people to, whether or not they take insurance, and what age groups they work with. (Speech-Language Pathologist, ID 214).*

*Maybe if there is an actual website that they can direct us as parents of where to go. (Mother to 3-year-old boy, ID 136).*

Both also suggested the need for evidence-based parent-focused web resources that health professionals and parents could reference. While some health professionals discussed the benefits of having access to a dietitian on consult for professional advice, many expressed difficulties in referring patients to dietitians due to a lack of awareness of local availability, with some desiring more physical nutrition resources on hand (e.g., handouts, pamphlets) to provide to families when a referral to a dietitian is not possible. Moreover, parents suggested that more community-based offerings like feeding or food play groups, even informal ones, should be made available to support meaningful connections to other families facing similar struggles.

*I do believe they should have eating groups and that there should be groups in my town of people like, you know, getting their kids together to try new foods and play with food. (Mother to 9-year-old boy, ID 107).*

## 4. Discussion

Our qualitative study revealed that while health professionals and parents of autistic children shared some perspectives on nutrition-related challenges and care for children with ASD, they also had distinct viewpoints. While both groups acknowledged the need for tailored support from an interdisciplinary care team, having reasonable expectations, and access to autism-specific educational resources and referrals, key themes arose which highlighted a mismatch in priorities between the two groups across several domains.

### 4.1. Addressing nutrition-related challenges

It was evident that both health professionals and parents of autistic children were motivated to address the numerous nutrition-related challenges faced by children on the spectrum. These findings are encouraging given that nutritional deficiencies, overweight, and obesity are a concern for children on the spectrum and can significantly negatively impact a child’s physical, emotional, and cognitive well-being if left unaddressed (12). While health professionals tended to prioritize weight imbalances or complex medical nutrition issues, parents emphasized upstream challenges that impacted their child’s quality of life, including food selectivity and sleep disturbances affecting appetite.

Selective feeding emerged as a key theme among parents, even if their child was experiencing typical growth. Selective eating can lead to meal-related anxiety, social isolation, and conflicts during family meals (27, 28). Parental attempts to manage selective eating may also lead to more obesogenic feeding practices, such as using screens to promote consumption, using food as bribes or rewards, and an increasingly limited diet of heavily processed foods (29, 30). Likewise, poor sleep is linked to overweight and obesity among children (31), but few professionals identified it as a concern to address, contrasting with nearly all parents who cited it as a priority concern. Even when a child is within a healthy weight range, lack of sleep over time can increase the long-term risk of overweight or obesity.

### 4.2. Barriers and facilitators to care

#### 4.2.1. Motivation

While some health professionals perceived parents as lacking motivation or the ability to make changes, parents felt that health professionals themselves lackthe ed knowledge and expertise to take concerns seriously, thereby affecting their own motivation. In the newly released clinical practice guidelines for overweight obesity, the American Academy of Pediatrics recognizes that tailored advice is often deficient in healthcare for children with disabilities, which can widen health disparities (32). The guidelines recommend using motivational interviewing as a patient-centered approach to behavior change.

Motivational interviewing requires healthcare professionals to identify and reinforce the patient/parent’s motivation to change when working with families (32). Suppose professionals are better aware of parent motivations. In that case, they may be able to facilitate more productive and collaborative strategies for addressing nutrition-related challenges when children are not yet ready to engage in decision-making. In our study, some parents expressed hesitance in discussing food-related issues because they felt that health professionals passed judgment when their children had selective diets or ate unhealthy foods. Motivational interviewing techniques, including non-judgmental, open-ended approaches and simple affirmations of challenges, may be positive and effective ways to engage in critical nutrition-related conversations with parents.

Another way to potentially address motivation-related challenges may be to educate providers about health disparities and practice limitations faced by autistic children. Research shows that using data to highlight practice deficiencies with providers can be one way to promote more family-centered care (33). A recent meta-synthesis of parent experiences with healthcare for their autistic children suggested that some medical providers are unaware of what they do not know about autism, which can lead to communication barriers and inadequate healthcare delivery (34). One concrete recommendation from the study was for providers to allow parents to be more active in contributing their expertise about their child during visits.

#### 4.2.2. Capability

The lack of autism-specific nutrition-related knowledge was a clear barrier for professionals and parents. Parents often perceived health professionals’ lack of addressing nutrition challenges or growth concerns as indifference, but the professionals shared that they felt ill-equipped to offer meaningful solutions for their autistic patients who faced unique behavioral and sensory challenges. In our study, most allied health professionals reported inadequate knowledge and training on nutrition needs for children with autism and felt uncertain about when to refer or provide care. Unfortunately, research shows that children with developmental disabilities such as autism often do not receive needed referrals for specialists (35).

In most cases, pediatricians are the primary medical contact for parents and are often perceived as the most authoritative and credible source for health-related information, including nutrition (36). However, not all general pediatricians are comfortable discussing ASD treatment options with parents due to a lack of knowledge about autism (37), even leading some pediatricians to discourage treatment or necessary therapies, which can lead to increased challenges for children as they transition into adulthood (38). In our study, multiple pediatricians acknowledged that their medical training did not emphasize nutrition. Therefore, pediatricians may benefit from additional autism-focused training, especially concerning nutrition care.

Our findings suggest that interdisciplinary healthcare professionals could also benefit from training on identifying nutrition-related risks, providing basic nutrition education, and making appropriate referrals as needed. The National Research Council Committee on Nutrition in Medical Education and the Institute of Medicine recommend a minimum of 25–30 h of nutrition education for medical professionals to understand nutrition concepts. Previous research has shown that medical professionals can increase their nutrition knowledge by participating in a 2-credit-hour elective course in clinical nutrition or a 1.5-credit-hour course on nutrition and lifestyle modifications during an academic year (39). Basic training on promoting healthy lifestyle habits, including physical activity, sleep, and screen use, could also be beneficial across disciplines.

#### 4.2.3. Opportunity

Our study found that despite encountering nutrition-related challenges regularly, few health professionals reported using regular diet/lifestyle screening for their patients with autism. Recommendations published by the American Academy of Pediatrics in 2020 propose that autistic children receive regular assessment of both growth and nutrition/lifestyle factors, regardless of their weight status (40). The guidance also recommends assessing many of the nutrition-related challenges our participants identified, including sleep, gastrointestinal problems, and psychotropic medications.

Although screening is important, it is not enough to meet the needs of patients who require more guidance. According to a position paper published by the Academy of Nutrition and Dietetics, nutrition services conducted by registered dietitian nutritionists are an essential element for effective interdisciplinary care of children with developmental disabilities like autism (41). However, many professionals and parents in our study reported limited resources related to nutrition services. Several medical professionals expressed the need for a list of local specialists, such as registered dietitians, to whom they could refer patients for nutrition care assistance. Others indicated the need for a better understanding of how and when to trigger referrals and what red flags to look for that would warrant more support for a child. Further research could explore the impact of basic nutrition-related screening and intervention in interdisciplinary settings.

### 4.3. Education opportunities and resource availability

Healthcare professionals and parents of children with autism desire more autism-specific educational resources on websites and handouts related to dietary guidance, food selectivity, dietary supplements (such as probiotics and fiber), gut health, and pica. The availability of nutrition-related resources to the public may give parents access to knowledge, even when medical professionals cannot provide it. Consistent with other literature on parents of children with neurodevelopmental disabilities (16), parents in this study identified other parents of autistic children as an essential source of nutrition information when specialists were unavailable and desired more community resources and opportunities to connect with other parents using evidence-based materials. Notably, our participants rarely discussed physical activity despite its essential role in growth, mental health, and appetite (25). This could be due to a lack of awareness or lack of community resources. Inclusive opportunities for physical activity are often lacking for children with autism and other developmental disabilities (16), thus leading to increased levels of physical inactivity among this population (19).

### 4.4. Implications

This qualitative study is one of the first to compare attitudes toward nutrition care for autism between interdisciplinary professionals with diverse practice backgrounds and parents of children with ASD. One strength of the study was the ethnically diverse group of parent participants, including those with autistic girls who are less studied and fathers who are often excluded from feeding and parenting research (19, 42). However, the generalizability of our findings may be limited as our sample focused on health professionals and parents in California, and access to services may vary regionally. In addition, our data were collected between 2019 and 2020 and may not reflect changes in healthcare delivery and attitudes toward nutrition and dietary behaviors during the COVID-19 pandemic. Finally, the study was conducted only in English, limiting perspectives of non-English speaking families. Future studies could assess a broader range of parents and healthcare professionals across regions, races, and ethnicities, and levels of support needs for children with autism.

## 5. Conclusion

Our qualitative study found that while healthcare professionals and parents of autistic children shared some perspectives on nutrition-related challenges and care, there were also significant points of divergence that require further exploration. We found that while interdisciplinary health professionals who serve autistic children were motivated to provide care, many face challenges in accessing nutrition-related knowledge and resources. Parents often perceived providers to lack concern about nutrition-related challenges, which could be more due to a lack of resources than motivation. In these cases, nutrition-related challenges could be addressed by an interdisciplinary team or through referrals, though few professionals described referring out to registered dietitians. Both groups expressed interest in accessing autism-specific resources for education, referral, and screening guidance. Future research could explore developing healthcare training models that improve the competency of health professionals in providing nutrition care and promoting evidence-based guidance for children with ASD. These findings underscore the need for more integrated approaches to nutrition care for autistic children, which could ultimately improve their overall health and well-being.

## Data Availability

The raw data supporting the conclusions of this article will be made available by the authors, without undue reservation.

## Footnote

How we talk about autism can reflect and reinforce ableist beliefs, as shown in the growing body of literature (43, 44). We recognize that there are differing opinions among the autistic community regarding preferred language and terminology (45, 46). Therefore, to show respect for these varying perspectives, both person-first (e.g., a child with ASD) and identity-first (e.g., an autistic child) language will be used.
