# Supplementary material for: Priorities, barriers, and facilitators for nutrition-related care for autistic children: a qualitative study comparing interdisciplinary health professional and parent perspectives
Source: Front Pediatr. 2023 Aug 15;11:1198177. doi: 10.3389/fped.2023.1198177 (PMC10465129; doi:10.3389/fped.2023.1198177)
Supplement: Supplementary file 1 [file Table1.pdf]

**Supplementary Table 1. Barriers and facilitators to care for nutrition-related challenges for autistic children**

| **Theme: Barriers to care for nutrition-related challenges** | | |
|---|---|---|
| Shared subthemes: health professionals and parents | Illustrative quotes | Domain [a] |
| Competing priorities for families | But honestly, we had other more behavioral concerns to address. It just wasn't a priority to me. (Mother to 10-year-old boy, ID 119)<br><br>So what happens is a lot of the things can be lost in translation because the parents are tired. They have a lot going on. They work, they have the kids. (Registered dietitian nutritionist, ID 217)<br><br>Probably parents trying to pick their battles. Mealtime is a very big hot button for parents, especially when children are young and difficult and don't want to eat. (Speech-Language Pathologist, ID 214) | Motivation |
| Lack of autism-relevant information | With autism specifically, I think it's difficult because even if we have nutrition classes or supplemental information for the parents to look into, very rarely are they specifically for autistic kids. (Pediatrician, ID 227) | Capability |
| Providers unsure how to help | Most of us didn't get much nutrition education in medical school or residency. (Pediatrician, ID 225)<br><br>But I haven't gotten much help from the developmental pediatrician, I haven't gotten much from his developmental psychologist. Even though I bring up these issues, they have really no big suggestion. (Mother to 7-year-old boy, ID 118)<br><br>When it comes to more neurological, developmental, it's not that they're not concerned, but they feel like it's not their expertise and, like "hey, someone else should take over this." And I don't know if they always feel comfortable referring you to the next person, right? So our pediatrician never did. (Mother to 4-year-old boy, ID 113)<br><br>They sent me to a dietitian when it first started all happening. It was for adults, so this person didn't even really know. There just no specialist for kids on the spectrum. And no, I don't think it's taken seriously. (Mother to 9-year-old boy, ID 107) | Capability |

| | | |
|---|---|---|
| Lack of access to nutrition services | There's definitely a lack of nutrition and feeding therapy services available, both privately and through the Regional Center covered care services [California state services]. Even if parents are willing and wanting to put in the work, a lot of times there's not the service available to guide them. (Pediatrician, ID 222)<br><br>I can't actually have a dietitian on our team, unless our doctor refers us, because of our medical insurance. (Mother to 11-year-old boy, ID 100)<br><br>Well, I haven't been referred to one [registered dietitian nutritionist]. I wasn't even offered one. (Mother to 7-year-old boy, ID 118) | Opportunity |
| Limited time during visits | A lot of us already have large scopes of practice and it's like that's such an important aspect and yet we just don't have time. (Speech-Language Pathologist, ID 218)<br><br>With pediatricians, and I get it that they're super busy, but like you give them the form [intake about child's food habits] and they don't really read it with you. They kind of skim it. (Mother to 3-year-old boy, ID 104) | Opportunity |
| Difficult doing nutrition education with child present | Having an autistic child with you at the pediatrician's office, I don't feel like you have the time to really focus on your answers. I'm not going to retain much because my brain has just been in fight or flight at that point, I'm like, "just get me home and out of this place!" (Mother to 4-year-old boy, ID 128)<br><br>I can give you many examples where I'm with the mom and the teenage son in the room . . . and I'm trying to do an interview and you can't even really get in a good consult because there's so much distraction there. (Registered Dietitian Nutritionist, ID 216) | Opportunity |
| Family financial limitations | A lot of my families too, who are very low income… in not having control or influence on what a kid eats and whether or not they're hungry, that can change the way an entire session with the child will go or an entire day a child will have. (Board Certified Behavior Analyst, ID 219)<br><br>So, some of it is the resources of just having the money to buy the food or to maybe screen different foods, right? You know "Hey, why don't you go buy this, this, this, and this?" | Opportunity |

| | | |
|---|---|---|
| | That's been a problem before, definitely. (Board Certified Behavior Analyst, ID 220)<br><br>And there's just no good options. A working mom, working single mom to [be able to] give him unprocessed foods that have plenty of nutrition and protein. (Mother to 7-year-old boy, ID 118) | |
| Subthemes among health professionals | Illustrative quotes | Domain [a] |
| Lack of parent compliance | I noticed that it's the parents who have a busy schedule that don't really have the time to create that eating structure to begin with. (Occupational Therapist, ID 224)<br><br>I would say another barrier, but this is just typical for any type of parent training program is having a hard time to make sure that parents are actually continuing with the programming when sessions are over. (Board Certified Behavior Analyst, ID 230) | Motivation |
| Change may not be possible | If we were trying to increase activity or we were trying to change intake to foods that were lower in calories or healthier choices, it was one thing to recommend it, and it was another thing to actually have it take place. Mainly because the child would be unwilling to do it. (Pediatrician, ID 225)<br><br>I also see kids that will very repetitively eat the same thing all the time, and I know that it's unhealthy for them instinctually, but it's hard to do anything about it because they're so entrenched in their schedule and their diet. (Occupational Therapist, ID 223) | Motivation |
| Concerns that intervention may worsen eating challenges | We can do a lot of harm if you're forcing a child to eat a food and you're not doing it carefully enough and it makes the issues even worse. (Board Certified Behavior Analyst, ID 219)<br><br>Parents are afraid that if they don't give the kids what they know that they'll eat, that the kids will not be gaining weight. That further restricts their diets or further narrows their diets (Pediatrician, ID 222)<br><br>I think the concern for a lot of parents, too, is like they're wary of working on it because they don't want their kiddo to like stop eating everything altogether. And they're eating | Motivation |

| | | |
|---|---|---|
| | really unhealthy foods, but at least they're eating you know. (Board Certified Behavior Analyst, ID 221) | |
| Limited referral options | There's very few opportunities to refer to a, just a dietitian. Developmental Pediatrician, ID 234) | Opportunity |
| Subthemes among parents | Illustrative quotes | Domain [a] |
| Concerns not taken seriously | Because one, most doctors, and I hate to say it like this, but can be very condescending to a patient's requests or concerns. Yeah, they see the child for five minutes in the appointment, but we as the parents live with the child 24/7. The parent input is very important, and the concerns need to be addressed. (Father to 9-year-old boy, ID 144)<br><br>The general pediatrician has not been very receptive. He tends to brush these things [feeding challenges] off. (Mother to 6-year-old boy, ID 109) | Motivation |
| Judgment from providers | Sometimes I feel that in discussions on picky eating, they [health professionals] tend to be kind of like judgmental and it falls hard on the parent. And the focus is on us, and our children sometimes are unable. Sometimes they cannot control the gagging reaction or sometimes they cannot control the aversion to a texture. (Mother to 6-year-old boy, ID 109)<br><br>If you don't live with it, it's tough to understand it on a daily basis. (Father to 9-year-old boy, ID 144) | Motivation |
| Child's growth in normal range | His doctors, they don't seem too concerned because he's not losing weight (Mother to 13-year-old boy, ID 135)<br><br>So, I just been getting a lot of run around, so I've been researching on my own. But, it hasn't successful. But I haven't felt a lot of support from the nutrition part because they think he is good cause he's at a good weight. (Mother to 3-year-old boy, ID 104) | Capability |
| Unaware of how to navigate services | I didn't know what to do, or I didn't know what was the next step to do to help my child get the help he needed. (Mother to 6-year-old boy, ID 127)<br><br>A lot of moms or parents are going through [feeding issues] and we all share the same thing and we're at a loss. And at school, they don't really focus on that [eating] as much because they're mostly focused on academics. So, it's hard to get assistance. (Mother to 3-year-old boy, ID 104) | Capability |

| Theme: Facilitators to accessing care for nutrition-related challenges | | |
|---|---|---|
| Shared subthemes: health professionals and parents | Illustrative quotes | Domain [a] |
| Individualized support | I would say first and foremost, just making sure that I'm taking into account the family's culture, so what their daily routine is like, what their menu, what type of food that they're usually eating and then making sure that the interventions and strategies that I teach and educate the parents with can easily fit it to their everyday routine. (Occupational Therapist, ID 224)<br><br>I think it's like we need to be more customized to each parent and let them know not all kids have the same issues. (Father to 9-year-old boy, ID 144) | Capability |
| Access to an interdisciplinary team | Oh, the registered dietitians on our team for sure. Yeah, I'm really lucky that we have RDs in house, cause I definitely feel very lost without them. (Occupational Therapist, ID 211)<br><br>So, we have a team of therapists. This year, because of his diagnosis, we now have a developmental pediatrician we can contact. (Mother to 6-year-old boy, ID 109)<br><br>Because eating is a behavior, but it's also internal and it's also psychological and it's also oral motor. So, there needs to be like a really good cohesive, comprehensive team that's there to support the child in feeding. (Board Certified Behavior Analyst, ID 219) | Opportunity |
| Subthemes among health professionals | Illustrative quotes | Domain [a] |
| Realistic expectations | I think that trying to break it down for parents usually makes it less daunting and makes them more able to grasp and practice what it is we talk about in the office at home. (Pediatrician, ID 222) | Motivation |
| Embracing slow progress | I would say a very, very detailed treatment plan of slow, slow progression. (Board Certified Behavior Analyst, ID 220)<br><br>Definitely flexibility, patience, the ability to go in at a pace and take baby steps and not have expectations that are out of line with what the parent feels they can do, being able to | Capability |

| | | |
|---|---|---|
| | translate what their recommendations, how their recommendations will show up for the child. (Speech-Language Pathologist, ID 214) | |
| Nutrition-related screening part of practice | Part of that initial assessment when we hear about food restrictions, food rigidity or any other concerns about if they have any other concerns about growth (Developmental Pediatrician, ID 233)<br><br>Every initial assessment that we do, we do specifically ask for an adaptive skills history and part of that includes you know feeding both from a nutrition standpoint you know what is their diet like, do they get a well-balanced diet and then also just from a self-skills standpoint are they able to feed themselves, are they able to you know use utensils, pick out their own food, you know choose an appropriate diet you know depending on the age of the child and their developmental level. (Pediatrician, ID 235) | Opportunity |
| Subthemes among parents | Illustrative quotes | Domain [a] |
| Other parents of autistic children | It's a lot easier to talk one parent to another than a parent to a medical doctor sometimes. (Father to 9-year-old boy, ID 144)<br><br>Nothing helps outside other than the mommy group talks that we ask each other like what works for your kid. And that’s been helpful. But it’s super common among all our moms that our kids don’t want to eat and have a hard time. (Mother to 3-year-old boy, ID 104) | Motivation |
| Mental health professionals | I've gotten the most feedback and understanding from a neuropsychologist. Somebody that has a little bit more understanding of the nervous system itself, and sensory relation. And not just, Oh it's anxiety. Or, “He'll outgrow it.” Or,,“Keep trying.” (Mother to 11-year-old boy, ID 100)<br><br>One of the psychiatrists he was seeing actually recommended that because I was giving him like a Pedisure and he's like, he's getting bigger. She says give him this because it has more carbs and more protein and that's what he needs. (Mother to 13-year-old boy, ID 135)<br><br>She [psychologist] actually did recommend us to read the book regarding nutrition and how it affects ADD and autism. (Mother to 4-year-old boy, ID 128) | Capability |
| ABA | Well when we did feeding therapy, we did it with ABA, which I think they're most qualified for getting food in its system. (Mother to 6-year-old boy, ID 132) | Capability |

| | | |
|---|---|---|
| | Well, in ABA, they're working on him. They're working on trying new foods and, and stuff like that. Different textures of foods sort of like peanut butter and stuff like that. (Father to 7-year-old boy, ID 130) | |

[a] Based on the Theoretical Domains Framework of behavior change
