# Supplementary material for: Priorities, barriers, and facilitators for nutrition-related care for autistic children: a qualitative study comparing interdisciplinary health professional and parent perspectives
Source: Front Pediatr. 2023 Aug 15;11:1198177. doi: 10.3389/fped.2023.1198177 (PMC10465129; doi:10.3389/fped.2023.1198177)
Supplement: Supplementary file 2 [file Table2.pdf]

Supplementary Table 2. Themes with illustrative quotes regarding desired educational topics and resources related to nutrition-related challenges for autistic children

**Theme: Desired topics of interest for education related to nutrition-related challenges**	
Shared subthemes: health professionals and parents	Illustrative quotes
Dietary guidelines specific to autism	I know every child is different, and the nutritional needs are different, I would like to have some guidelines, maybe some standards for age groups. (Mother to 6-year-old boy, ID 109)<br><br>I think it would be good for them [health professionals] to give us information on what is nutritionally required, the bare minimum because a lot of our kids are not getting a lot of what is required of nutrition. (Mother to 6-year-old boy, ID 132)<br><br>Yeah honestly, the dietary factors where you’re mentioning following a restricted diet for health, religious, or ethical purposes yeah, I don’t think we get a lot of formal education about that. (Pediatrician, ID 235)<br><br>I guess the dietary factors . . . I know that kids with autism have problems with the texture and eating a variety of foods, but I don’t know much about how we can change that. (Pediatrician, ID 227)
Strategies to address food selectivity	Ok so we identify this thing, what can I do? I think in my child, a lot of it falls within what do I do to address the rigidity in food choice? (Mother to 6-year-old boy, ID 109)<br><br>I think it’s frustrating to parents that deal with food rigidity because that impacts their ability to go out, socialize, go to restaurants, things like that. (Developmental Pediatrician, ID 233)<br><br>I think in terms of picky eating, especially for myself, because you know it’s one of the areas that we work on in feeding therapy. So, techniques for getting kiddos to eat a variety of foods. (Speech-Language Pathologist, ID 218)<br><br>Basically, how have other parents gotten around these issues, and how do they get their kids to eat vegetables and meat and increase their food choices. (Mother to 7-year-old boy, ID 118)<br><br>How to go about introducing new foods . . . It just seems like a lot of parents feel so helpless on how to get them [their child] to try new things out without bribing them. (Mother of son with ASD; ID 135)

Dietary supplements	“A lot of parents are doing that kind of intervention. They refer to it as biomed. You know, are they leaning towards supplements and natural remedies or leaning towards psychiatric or a combination of both.” (Mother to 4-year-old boy, ID 113)<br><br>Ways to get certain things naturally. You know, like let's say omega-3's, if your kid won't eat fish. I think things like that could be really helpful. (Mother to 6-year-old son, ID 110)<br><br>I have seen some families that do give quite a lot of supplements to their children with autism and in general I would say in, in the medical field we don't get a lot of training about the effects of like herbal supplements and you know how they interact with other medications or the impacts that they have on one's appetite. (Pediatrician, ID 235)
Efficacy of specialized diets for autism	A lot of diets have been proposed for these kids, or like supplements and stuff. Like, what is the deal with all these diets? Do these things work? (Mother to 5-year-old boy, ID 101)<br><br>Or specialized diets and supplements. A lot of parents come to me with that, with all sorts of different testing that's been done in all sorts of different ways. But I certainly don't have enough about know about to be able to advise them properly. (Pediatrician, ID 222)<br><br>There are a lot of families that have adopted some of the alternative therapies for autism that are not evidence-based. I have seen some families that say that they've had their child be on a gluten-free diet or casein-free diet or even keto diet because they feel like that might help with autism. . . I think is a, is a difficult issue and I don't think we get a lot of specific training on that. (Pediatrician, ID 235)
Subthemes among health professionals	Illustrative quotes
Autism	Most definitely in learning more about children on the autistic spectrum. I mean, most definitely more education about what that really is and how very different it could look for each child cause I believe medical professionals have kind of a very narrow view of what autism looks like. (Speech-Language Pathologist, ID 210)
Basic nutrition and feeding	Most of us didn't get much nutrition education in medical school or residency. They need to just continue to educate themselves about the behavioral aspects of feeding, and the restrictive behaviors, the sensory motor problems that kids have. (Pediatrician, ID 225)

Steps to take for providing support	Who's role is it to do the screening and to talk to them and tell them you know? I think that, sorry, there should maybe be some guidelines on how to, the steps to take that, after a screening been done, you know like you start feeding therapy first or you know or, do you see a nutritionist? I mean or do they need to get swallow studied? I mean, there's just so many things. (Registered Dietitian Nutritionist, ID 216)<br><br>I feel like oftentimes we can address it [nutrition concerns] but we would need access to a dietitian or knowing when we should go to them. (Occupational Therapist, ID 231)
Subthemes among parents	Illustrative quotes
Meeting dietary needs within a restricted diet	I would be most interested about how to balance their diet with things they'd eat. (Mother to 9-year-old boy, ID 107)
Gut health	. . . I know that yeast overgrowth can affect behavior, so more in depth about gut health. (Mother to 3-year-old boy, ID 105)<br><br>Maybe nutrition information for the gastrointestinal issues. I think that's a really big issues for a lot of people. (Mother to 6-year-old son, ID 110)
Pica	Maybe the topic of pica, you know if a child feels a compulsion to chew or eat anything that they're not supposed to. (Father to 3-year-old girl, ID 147)

**Theme 5: Desired resources to address nutrition-related challenges**	
Shared subthemes: health professionals and parents	Illustrative quotes
List of local specialists or providers to refer to	A list of local professionals that we can refer people to, whether or not they take insurance, and what age groups they work with. (Speech-Language Pathologist, ID 214)<br><br>. . . To have somebody to refer it [patients] to. In general, I think it's hard to find dietitians or nutritionists to refer to, but specifically for autistic kids . . . it might be more of a challenge. (Pediatrician, ID 227)
Parent-friendly website	Anything now where you can kind of click on a site, maybe techniques to get them to try different things, or things to try, like methods. (Mother to 10-year-old boy, ID 119)

	Maybe if there is an actual website or you know that that can direct us as parents of where to go. (Mother to 3-year-old boy, ID 136)<br><br>Having like a website or having some information for parents online for them to be able to read through and research through would be helpful to have all the information together that we think is clinically based. (Pediatrician, ID 227)<br><br>It could also include like suggestions for what could be done about it or provide links to additional resources or pamphlets or information things like that would be helpful too (Board Certified Behavior Analyst, ID 230)
Subthemes among health professionals	Illustrative quotes
Access to a registered dietitian nutritionist for professional advice	I think it would be incredibly helpful to have like a registered dietitian or nutritionist on the autism kiddos team. I think like having them educate us so we can educate parents would be fantastic or like direct education. (Speech-Language Pathologist, ID 218)<br><br>Have a list of local dietitians to reach out to if they [health professionals] need to ask questions (Board Certified Behavior Analyst, ID 230)
Evidence-based paper materials to supplement conversations	Maybe some sort of like complimentary handbook of recommendations with evidence-based recommendations or interventions based on the particular identified challenges. (Board Certified Behavior Analyst, ID 219)
Subthemes among parents	Illustrative quotes
Community groups	I do believe they should have eating groups, and that there should be groups in my town of people like, you know, getting their kids together to try new foods and play with food. (Mother to 9-year-old boy, ID 107)
