# Supplementary material for: Priorities, barriers, and facilitators for nutrition-related care for autistic children: a qualitative study comparing interdisciplinary health professional and parent perspectives
Source: Front Pediatr. 2023 Aug 15;11:1198177. doi: 10.3389/fped.2023.1198177 (PMC10465129; doi:10.3389/fped.2023.1198177)
Supplement: Supplementary file 3 [file Datasheet1.pdf]

Supplementary Material 1: Interview Guides

Excerpt from Health Professional Interview Guide

1. First, can you tell me a little about your background and the work that you do with children on the autism spectrum?
2. How often does feeding or nutrition come up during your work with patients?

Ok, moving onto the next part of our interview, I emailed you a brainstorm list of nutrition-and lifestyle factors that current literature has shown are potentially common or challenging for children with autism. Do you have that in front of you? [Wait for them to get it.]

List provided via .pdf to participants:

Possible Nutrition-Related Challenges for Children with Autism to Include in a Screening Tool

Growth
- Poor growth (recent)
- Rapid weight gain (recent)
- Overweight or obesity
- Underweight

Recent changes in intake
- Increased appetite
- Suppressed appetite
- Inability to sense hunger/fullness cues
- Excessive thirst
- Lower energy levels
- Gagging while eating

Dietary factors
- Diet consisting of majority highly processed carbohydrates
- Following a restrictive diet for health/religious/ethical purposes (e.g. gluten-free, casein free, specific carbohydrate diet, paleo, ketogenic, vegan)
- Limited dietary diversity (e.g. missing food groups, 10 foods or fewer, etc)
- Rigidity in food choice with self-imposed restriction for types of food, food presentation, temperature of food, food brand, or location (e.g. not eating at school or day care)
- Administration of numerous over the counter supplements (e.g. vitamins)
- Use of food rewards to reinforce behavior
- Use of foods (withholding) to punish bad behavior

Medical risks
- Pre-diabetes or diagnosis of diabetes (type 1 or type 2)
- Hyperlipidemia
- Family history of type 2 diabetes
- Food allergies/intolerances

Gastrointestinal issues
- Constipation

- Reflux

Medications that impact nutrition status (Antipsychotics, Stimulants, Anticonvulsants)

Quality of life
- Child expressing major anxiety/concern about diet
- Child expressing major anxiety/concern about growth/weight
- Parent expressing major anxiety/concern about diet
- Parent expressing major anxiety/concern about growth/weight

Lifestyle factor affecting growth or physical activity
- Poor sleep
- Excessive screen use
- Inactivity

3. Can you tell me which items you think are the most important to address for this population of children?
4. What barriers do you face in addressing nutrition care for children with autism?
5. Is there anything you have found to be especially successful when addressing nutrition-related care for children with autism?
6. In your mind, what resources do providers need in place to respond to concerns identified?
7. With respect to this list, are there any topics in particular that you or colleagues wished you knew more about?
    a. Probe: What about parents? What do you think they would care most about learning?
    b. Probe: Could you explain a bit why?

Excerpt from Parent Interview Guide

1. First, can you tell me a little about your child who is on the autism spectrum?
2. Have you had any eating or growth challenges that you've faced with [insert child's name]?
3. As of today, how do you feel about your child's eating or how he/she is growing?
4. In your opinion, of all possible options, who do you think is or has been most qualified to give nutrition advice for your child? Why?
5. Have you talked about these challenges with a pediatrician or other medical professional? Why or why not?
6. Can you talk about any other help or support that you've received for these challenges outside of the traditional medical community?

Ok, to start, I emailed you a brainstorm list of nutrition-related factors that current science has shown are potentially common or challenging for children with autism. This list is not a judgement on any parenting practices or decision about raising children. Do you have that in front of you? [Wait for them to get it.]

Great! Do you want to take a minute to look it over? We'll be talking about it for a bit.

List provided via .pdf to participants:

Possible Nutrition-Related Challenges for Children with Autism to Include in a Screening Tool

Growth

- Poor growth (recent)
- Rapid weight gain (recent)
- Overweight or obesity
- Underweight

Recent changes in intake

- Increased appetite
- Suppressed appetite
- Inability to sense hunger/fullness cues
- Excessive thirst
- Lower energy levels
- Gagging while eating

Dietary factors

- Diet consisting of majority highly processed carbohydrates
- Following a restrictive diet for health/religious/ethical purposes (e.g. gluten-free, casein free, specific carbohydrate diet, paleo, ketogenic, vegan)
- Limited dietary diversity (e.g. missing food groups, 10 foods or fewer, etc)
- Rigidity in food choice with self-imposed restriction for types of food, food presentation, temperature of food, food brand, or location (e.g. not eating at school or day care)
- Administration of numerous over the counter supplements (e.g. vitamins)
- Use of food rewards to reinforce behavior
- Use of foods (withholding) to punish bad behavior

Medical risks

- Pre-diabetes or diagnosis of diabetes (type 1 or type 2)
- Hyperlipidemia

- Family history of type 2 diabetes
- Food allergies/intolerances

Gastrointestinal issues

- Constipation
- Reflux

Medications that impact nutrition status (Antipsychotics, Stimulants, Anticonvulsants)

Quality of life

- Child expressing major anxiety/concern about diet
- Child expressing major anxiety/concern about growth/weight
- Parent expressing major anxiety/concern about diet
- Parent expressing major anxiety/concern about growth/weight

Lifestyle factor affecting growth or physical activity

- Poor sleep
- Excessive screen use
- Inactivity

7. Based on what is currently on this list, could you tell me, from your perspective, which things stand out as most important to address in a screening tool?
    - *Probe: Why did you choose these over the other potential options? What makes them most important in your mind?*
    - *Probe: Is this based on experience with your child? Other children?*
8. What else should be on this list?
9. Was there anything listed that did not seem to belong on this list at all?
    - *Probe: Anything seem repetitive or unnecessary?*
10. What would you personally be most interested in learning about if a pediatrician or therapist were going over a tool like this with you?
