# Supplementary material for: Priorities, barriers, and facilitators for nutrition-related care for autistic children: a qualitative study comparing interdisciplinary health professional and parent perspectives
Source: Front Pediatr. 2023 Aug 15;11:1198177. doi: 10.3389/fped.2023.1198177 (PMC10465129; doi:10.3389/fped.2023.1198177)
Supplement: Supplementary file 4 [file Datasheet2.pdf]

Supplementary Material 2 – Study Codebook

Node	Description
**Background (Both)**	
**1. Background (Parent only), Part 1**	***This parent node shouldn't have anything in it that's not in a child node***
Behavior or health challenges (non-feeding)	*Anything about non-feeding behaviors or health issues*
Child strengths	*Anything where the parent is talking about positive attributes of child ("he is good at", "he always…", etc)*
Family characteristics	*Code for household number – who does the child live with*
Story of dx	*Code about age of diagnosis and any relevant details*
Use of medication	Code any place where medication is discussed
**2. Background (Medical Professional Only), Q1 and Q2**	***This parent node shouldn't have anything in it that's not in a child node***
Work environment/patient demographics	*Code descriptions of where they work/their patient population*
Example topics/issues addressed with patients	*What types of issues are addressed, what their scope of practice is*
Frequency of nutrition coming up in work with patients	*Any mention of numbers, frequency of nutrition or growth challenges in their practice*

.

Node	Description
**Nutrition Care Experiences and Perceptions (Medical Professional Only)**	
**1. Barriers for providing nutrition care (Q3)**	***Things that prevented medical professionals from providing effective nutrition care***
Personal factor	*Mention of their knowledge, attitudes, and beliefs affecting nutrition care*
Behavioral factor	*Mention of their skills, practice, and self-efficacy affecting nutrition care*
Environmental factor	*Mention of their surroundings, social norms, institutional location, parental limitations, patient circumstances and peers affecting nutrition care*
Competing priorities	*Mention of having to choose between addressing different health issues or challenges, time-restraints*
**2. Facilitators for providing nutrition care (Q4)**	***Things that promote providing effective nutrition care from medical professionals***
Successful experiences	*Anything describing a success in assessment, intervention, and/or outcome*
Positive view of nutrition care	*Positive feelings or emotions related to nutrition care*
Use of a referral	*Mention of referring a patient out to another medical professional for further assistance*
Type of medical professional support	*Any support provided by the RD, pediatrician, developmental pediatrician, OT, ABA, SLP, etc.*
Other help or support	*Anyone else (parents, legal guardian, family members, teacher, friends) supporting the medical professional's intervention plan*
**3. Description of existing screening (Q8)**	***Any examples of current screening or lack of screening (code all)***

Node	Description
**NON-RD Medical Support Experiences and Perceptions (Parent only)**	
**1. Negative**	***Anything describing actual support they received in a negative way***
Barriers to getting support	*Things that prevented them from support, hypothetical or real (i.e. no one knows how to do this, money, etc)*
Negative view of medical support	*Anything describing actual support they received*
No support	*Indicated no support has ever been received*
**2. Positive**	***Anything describing actual support they received in a positive way***
Types of support providers	*Code all mentions of people or resources who have offered advice, medical care, or help*
Medical professionals - other	*Anyone other than a pediatrician or RD (i.e. OT, ABA, PT) that is in the medical field*
Other help or support	*Anyone else (i.e. teacher, friend, spouse, alternative medicine, etc)*
Pediatrician	*Any mention of a pediatrician*

Node	Description
**Tool Feedback (Both)**	
**1. Rationale for what is most important to include (the why)**	***You don't need to code every single thing they say as they go through the tool here (much of that will be in "Areas of Concern" below. Here just code the why they think whatever they mentioned was most important.***
**2. Descriptions of areas of nutrition or growth concern**	***Code all places where challenges with eating or growth factors (including sleep) are discussed as they list priority areas (i.e. poor growth, overweight, behaviors, sleep, etc). Be sure the WHY is coded above.***
Competing priorities	*Mention of having to choose between different health issues or challenges*
Overweight	*Any mention of overweight (even if parent just mentioned a doctor noticed)*
Sleep	*Mention of sleep*
**3. Parent-Only Concerns**	
Impact on self or family	*Description of impact of child's growth on the parents or family (not the child)*
Norm references - other kids or families' experiences	*Reference to "other kids with ASD" or "my child who is not on the spectrum", etc with respect to children or families.*
Impact on child	*Impact of challenges on the child (i.e. social, emotional, etc)*
No challenges	*Parent suggests no growth or eating issues. Code even if they discuss some later.*
Feelings about eating or growth (parent or child)	*This is any emotion (vs description) related to eating/growth. Must use feelings to code this. "It's frustrating." Or "I'm exhausted" or "It makes him feel depressed."*
**4. Provider-Only Concerns**	***Coding for the specific question about medication and diagnoses***
Medications	*Code all places where specific meds are mentioned*
Diagnoses	*Code all places where specific diagnoses are mentioned*

Node	Description
**Desired resources or information (Both)**	
**1. Topics of interest for resources or information**	***Anything topics participants would like resources/information to be on***
Feeding or picky eating	*Any discussion of feeding or "getting him to eat", etc information or resources*
Specific diets	*Mention of diets information or resources*
Supplements	*Mention of supplements information or resources*
Growth	*Mention of growth-related information or resources*
Sensory abnormalities	*Mention of sensory abnormalities-related information or resources*
**2. Populations needing resources**	*Code if participant specifically mentioned that a certain population needed education*
Parent-specific	*Mention topics that are needed specifically for parents*
Provider-specific	*Mention topics that are needed specifically for medical providers*
**3. Types of resources**	***Anything on the type of resource they would like information presented***
Website	*Mention of internet/web-based resources*
Book	*Mention of book resources*
Handout	*Mention of handout resources*
Pamphlets	*Mention of pamphlet resources*
Classes	*Mention of classes for information*
Focus Groups	*Mention of focus group for information*
